# Quantification of Functionalised Gold Nanoparticle-Targeted Knockdown of Gene Expression in HeLa Cells

**DOI:** 10.1371/journal.pone.0099458

**Published:** 2014-06-13

**Authors:** Meesbah Jiwaji, Mairi E. Sandison, Julien Reboud, Ross Stevenson, Rónán Daly, Gráinne Barkess, Karen Faulds, Walter Kolch, Duncan Graham, Mark A. Girolami, Jonathan M. Cooper, Andrew R. Pitt

**Affiliations:** 1 Institute of Molecular, Cell and Systems Biology, College of Medical, Veterinary and Life Sciences, University of Glasgow, Glasgow, United Kingdom; 2 Division of Biomedical Engineering, School of Engineering, University of Glasgow, Glasgow, United Kingdom; 3 Centre for Molecular Nanometrology, Department of Pure and Applied Chemistry, University of Strathclyde, Glasgow, United Kingdom; 4 School of Computing Science, University of Glasgow, Glasgow, United Kingdom; 5 Institute of Cancer Sciences, College of Medical, Veterinary and Life Sciences, University of Glasgow, Glasgow, United Kingdom; 6 Systems Biology Ireland and the Conway Institute, University College Dublin, Belfield, Dublin, Ireland; 7 Department of Statistical Science, University College London, London, United Kingdom; 8 School of Life and Health Science, Aston University, Birmingham, United Kingdom; Universidad de Castilla-La Mancha, Spain

## Abstract

**Introduction:**

Gene therapy continues to grow as an important area of research, primarily because of its potential in the treatment of disease. One significant area where there is a need for better understanding is in improving the efficiency of oligonucleotide delivery to the cell and indeed, following delivery, the characterization of the effects on the cell.

**Methods:**

In this report, we compare different transfection reagents as delivery vehicles for gold nanoparticles functionalized with DNA oligonucleotides, and quantify their relative transfection efficiencies. The inhibitory properties of small interfering RNA (siRNA), single-stranded RNA (ssRNA) and single-stranded DNA (ssDNA) sequences targeted to human metallothionein hMT-IIa are also quantified in HeLa cells. Techniques used in this study include fluorescence and confocal microscopy, qPCR and Western analysis.

**Findings:**

We show that the use of transfection reagents does significantly increase nanoparticle transfection efficiencies. Furthermore, siRNA, ssRNA and ssDNA sequences all have comparable inhibitory properties to ssDNA sequences immobilized onto gold nanoparticles. We also show that functionalized gold nanoparticles can co-localize with autophagosomes and illustrate other factors that can affect data collection and interpretation when performing studies with functionalized nanoparticles.

**Conclusions:**

The desired outcome for biological knockdown studies is the efficient reduction of a specific target; which we demonstrate by using ssDNA inhibitory sequences targeted to human metallothionein IIa gene transcripts that result in the knockdown of both the mRNA transcript and the target protein.

## Introduction

Gene therapy has become a key focus of biomedical research, as a potential treatment for numerous genetic diseases, including cancer [1]–[2]. However, the translation of this technique into clinical practice has been limited by the low efficiency of therapeutic agent delivery [3]. The most common approach to delivery *in vitro* involves the use of recombinant viruses as gene carriers due to their high transduction efficiencies, which can result in high levels of protein expression [4]. However their use *in vivo* has been hampered by the fact that many of the viral proteins trigger strong immune responses and scaling up recombinant virus-based delivery systems remains challenging [5]–[7].

Non-viral gene delivery systems, including cationic lipids, polymers, dendrimers, and peptides [8]–[12], show significantly reduced transfection efficiencies compared to the viral systems. Recently, a new avenue of research has focused on nanoparticles as delivery vehicles. Careful engineering of their surface properties with specific recognition elements such as antibodies has provided an ability to target specific cells (particularly cancer cells) [13]–[14].

In fact, vectors based upon a variety of nanoscale carriers, including carbon nanotubes, iron oxide, silica, and gold nanoparticles have all demonstrated successful gene delivery [15]–[20]. Gold nanoparticles are of particular interest as they are biologically inert, which by implication suggests that they should not be cytotoxic [21]. They are easily synthesized and, as stated, are readily functionalized using established thiol chemistries, enabling the engineering of the surface with receptors (or indeed more subtle changes in the physico-chemical properties). A complex picture has now emerged within the literature, where nanoparticle biocompatibility can be seen to be dependent upon dose, cell type and surface properties. For example, while no toxicity has been found in studies using gold nanoparticles in BHK21 and HepG2 cells, in others cell lines such as A549, the opposite is true (evidenced by concentration-dependent morphological changes, as well as decreased cell viability, as the gold nanoparticle concentration increased [19], [22]–[24]).

The fact that the surface of gold nanoparticles offers well established routes for functionality makes them appealing vehicles. Charged or hydrophobic motifs can be readily bound to the surface, and indeed can be combined with modification protocols that include targeting moieties such as antibodies and receptors. These too can be mixed with a payload such as double-stranded DNA (dsDNA) or single-stranded DNA (ssDNA), which is then transfected into cells [25]–[29]. In one example, Rosi *et al*. [29] demonstrated the knockdown of genetically-encoded enhanced green fluorescent protein (EGFP) after transfection with ssDNA-functionalized gold nanoparticles by measuring fluorescence of the residual target protein in the cell. In a more recent study, Kim *et al*. [3] were able to demonstrate the knockdown of p53 protein in HeLa cells using ssDNA-functionalized gold nanoparticles.

Nanoparticles have been shown to enter cells in one of two ways, either by passive uptake, possibly diffusion [30], or by endocytosis [31]. In the latter case, the particles migrate through the cells via a variety of different vesicles to reach the nucleus [32]–[33]. The different transfection reagents that have been developed to facilitate their uptake either by a passive or active mode, influence not only the efficiency of transfection, but also the localization of the payload after it enters the cell, leading to a complex array of factors that needs to be fully understood to inform a rational strategy if these agents are to be used in gene therapy.

Here we compare the effect of different transfection reagents on the insertion and localization of ssDNA-functionalized gold nanoparticles targeted at metallothionein-IIA (MT-IIA), in HeLa cells. Metallothioneins are a family of ubiquitous low molecular weight proteins that have well-established regulatory roles in the cell [34], making them a target of choice for applications in metal ion homeostasis, in the detoxification of heavy metals and as protective cellular stress proteins [34]–[35]. They also play an integral role in cell survival via their interaction with the transcription factors NF-κB and p53; and elevated metallothionein levels have been found in a number of cancers [36]–[38]. MT-IIA accounts for approximately 50% of the total cellular metallothionein protein [39], and is induced by a wide variety of environmental stresses and agents [34]–[35], [40]. Here, we used cadmium to manipulate the levels of the MT-IIA in the cell, to calibrate gene silencing effects (Karin *et al*. [39]).

We now describe a method to quantify the gene silencing properties of small interfering RNA (siRNA), single-stranded RNA (ssRNA) and ssDNA, both free and bound to nanoparticles. We show that ssDNA-dependent knockdown of gene expression using functionalized gold nanoparticles occurs at the mRNA level and results in the loss of the specific mRNA transcript as well as the target protein. This approach to gene knockdown is particularly attractive as it bypasses both translation and post-translational mechanisms. In addition, by targeting mRNA, it also avoids the risk of non-specific gene knockdown.

Our study also clearly demonstrates the importance of appropriate reagent selection and validation. In testing three different transfection reagents, we observed varying transfection efficiencies but more importantly, we detected biological artefacts present in HeLa cells that were treated with Lipofectamine 2000 or Matra but not in those that had been treated with GeneJuice.

## Methods

### Gold nanoparticle synthesis

Gold nanoparticles were prepared by the Turkevich method [41]. Briefly, HAuCl_4_ was reduced using sodium citrate to yield colloidal gold with a diameter of approximately 30 nm. To prepare functionalised nanoparticles, 20 µL of oligonucleotide (5′-thiol-3′-fluorescein isothiocyanate (FITC); prMJ514 441 pmol/µL or prMJ515 415 pmol/µL) was added to 1 mL citrate-reduced Au colloid (17 nM) and incubated for 3 h. Phosphate buffer (60 mM, pH 7.4) was added to a final concentration of 10 mM and then samples were salt-aged (NaCl, 2 M) to a final concentration of 0.05 M over a period of 24 h. Samples were left for 1 h after the final salt addition and then centrifuged at 5000 rpm for 20 min, the supernatant was removed and samples were re-suspended with 0.05 M phosphate buffer saline (PBS); the washing process was repeated a further two times. Nanoparticles were resuspended to a final concentration of 0.5 M in PBS and stored at 4°C. Functionalized gold nanoparticles were stable at 4°C for up to 2 weeks, however a reduction in the intensity of the FITC fluorescence signal was observed over this period of time due to the deterioration of the FITC molecule.

In order to characterize nanoparticle size, functionalized gold nanoparticles were diluted appropriately in dH_2_O to a total volume of 1 mL and then analysed by dynamic light scattering using a Zetasizer Nano ZS (Malvern Instruments). Three measurements were made for each sample and each measurement represents the output of 20 runs (Figure S1).

### General Culture Conditions

HeLa cells (ATCC number CCL-2) were maintained in Dulbecco's Modified Eagle Medium (DMEM) supplemented with 4 mM L-glutamine and 10% FBS at 37°C in an atmosphere that contained 5% CO_2_.

### Transfection and treatment of HeLa cells for hMT-IIa and hMT-IIA knockdown studies

For each transfection, the required concentration of siRNA, ssRNA or ssDNA (Eurofins MWG, Table S1) or ssDNA-functionalized nanoparticles were transfected into 2×10^5^ HeLa cells using GeneJuice (Novagen) as recommended by the manufacturer's instructions. Transfected cells were incubated for 16 hours and subsequently induced with 12.5 µM CdCl_2_ for 4 hours before mRNA and protein analysis.

### RNA purification, cDNA synthesis and qPCR

Total RNA was prepared using the miRNeasy mini kit (Qiagen). mRNA was isolated using Dynabeads mRNA Purification Kit (Invitrogen) and was reverse transcribed using Superscript II enzyme (Invitrogen). qPCR analysis was carried out in a Lightcycler 480 (Roche) using Lightcycler Probes Master mix (Roche). The nucleotide sequences of the primers used in each reaction are shown in Table S1. Total *hMT-IIa* gene expression was measured relative to the expression of *B2M* in control sequence or *hMT-IIa*-specific sequence transfected cells. *B2M* is a stably expressed chromosomal gene for beta-2-microglobulin [42].

### Protein analysis

Cell lysates were quantified using the Quant-iT protein assay kit (Invitrogen), and 1 µg protein was separated on 4–12% Tris-Acetate acrylamide gels. The protein bands were then transferred to an Immobilon-P membrane by electroblotting. Metallothionein (Dako; M0639) and α/β-tubulin (Cell Signalling; 2148) antibodies were used for Western blotting which was performed using standard conditions. Signal was detected with SuperSignal West Pico Chemiluminescent substrate (Thermo Scientific) using the GBOX/CHEMI-HR16-E-BOX Gel Documentation System (Syngene) and analysed with the GeneSnap software (Syngene). Membranes were stripped of antibodies and then re-probed for the control protein (α-tubulin) to verify equal protein loading. Western blot images were analyzed with ImageJ (http://rsb.info.nih.gov/ij).

### Microscopy

2×10^5^ HeLa cells were settled on glass coverslips for 2 h. These cells were transfected with 0.2 or 1 nM FITC-NPs in the presence and absence of Matra (IBA), Lipofectamine 2000 (Invitrogen) or GeneJuice (Novagen). After 16 h, the cells were treated with 12.5 µM CdCl_2_ for 4 hours. Before visualizing the cells, coverslips were gently washed three times in PBS at 37°C for live cell microscopy (where a glass well was affixed to the coverslip so as to maintain the cells in PBS during imaging). For fixed cell microscopy, coverslips were washed three times in PBS for 5 minutes and this washing step was repeated after each of the following steps: cells were fixed in 4% paraformaldehyde for 20 minutes; permeabilized with PBS containing 5% BSA, 0.1% Triton X-100 for 5 minutes; blocked with PBS containing 5% BSA, 0.05% Tween 20 for 1 h; incubated with 2 µg/mL anti-LAMP1 (Santa Cruz; 18821) and/or anti-LC3A/B (Cell Signalling; 4108) in PBS containing 5% BSA, 0.05% Tween 20 for 16 h at 4°C; then incubated with 20 µg/mL anti-rabbit AF647 (Cell Signalling; 4414) and/or anti-mouse AF546 (Cell Signalling; 4409) in PBS containing 5% BSA, 0.05% Tween 20 for 2 h. Finally, cells were incubated in 1 µg/mL DAPI for 5 minutes, washed in PBS and then mounted using polyvinyl alcohol mounting medium with DABCO (Sigma; 10981).

Cells were imaged using either a Zeiss Axio Observer inverted microscope with an Andor LucaEM-R EMCCD camera and an x63, 0.75 NA objective, or a Zeiss LSM510 laser scanning confocal microscope with a x100, 1.45 NA objective. All images were acquired using the same exposure and detector settings for each spectral channel. Andor Solis software was used to acquire images from the Observer microscope, which were then adjusted so that, for each spectral channel, all images had an equal background-corrected electron count range. Color conversion and image overlays were performed using Corel Photo-Paint X3. Confocal image overlays were processed using Zeiss LSM Image Browser software.

For quantifying the fluorescence intensity from the FITC channel in live cells, ImageJ (http://rsb.info.nih.gov/ij) was used. A masking region was created by manually drawing around an individual cell in a phase contrast image. This mask was then used to measure the mean fluorescence intensity of the corresponding cell in a FITC image. Cells that appeared to be dying or dividing were not used for analysis. For all transfection conditions except Matra, measurements were acquired from >32 cells taken from 8 or more images. As there were far fewer surviving Matra cells, measurements for this transfection reagent were taken from 12 cells within 4 images.

### Statistical analysis of image data

All quantification experiments contained n = 4 biological replicates and experiments were repeated at least three times. The data was transformed simply by taking logarithms. The log_2_ transformed data were analyzed in JAGS [43], a Gibbs sampler for hierarchical models and CODA [44], a tool for examining Markov Chain Monte Carlo runs. A two-level Bayesian ANOVA model was used, modeling the intra-image variance at the first level and inter-image variance at the second level. The likelihood was specified as normally distributed, with an uninformative flat prior on the variance. The first-level was specified as normally distributed, with an uninformative inverse-gamma prior on the variance. The second-level mean was given an uninformative normal distribution.

The model calculated the posterior probability distribution over the mean of the ratio of the treated to untreated total fluorescence. The tails of this posterior distribution were compared to 1 (indicating no difference), to obtain a p-value. This p-value indicated the posterior probability that there was no difference in expression levels between the control and treatment samples. Hence, a lower p-value would indicate a greater likelihood that there was a difference between the control and treatment samples. 95% confidence intervals were also calculated for the mean ratio.

## Results and Discussion

### Characterization of the gold nanoparticles

As stated, the synthesis of gold nanoparticles was conducted using standard methods, as described by the Turkevich method [41]. The citrate salt acts as a reducing agent and forms a layer of negatively charged citrate ions over the surface of the gold nanoparticle, so inducing sufficient electrostatic repulsion to generate uniform and generally spherical nanoparticles [45].

Endocytosis of gold nanoparticles is size dependent. In a study in HeLa cells, Wang *et al.* [46] have previously demonstrated that smaller nanoparticles (up to 40 nm) are internalized by endocytosis whereas larger nanoparticles (70–110 nm) collect at the cellular surface. In our hands, the mean diameter of the synthesized gold nanoparticles prepared in this paper was measured to be approximately 30+/−10 nm (Figure S1) suggesting that the most likely mechanism by which nanoparticles would be expected to enter the cell was by endocytosis.

The surface of gold nanoparticles can be modified with a wide variety of ligands including amine, carboxylate, isocyanide, phosphine, and thiol groups [47]–[53]. In this study, gold nanoparticles were modified with thiol-linkers and FITC-tagged ssDNA sequences (5′-SH-ssDNA-FITC-3′) to make use of the strong covalent bond that forms between gold and sulphur [54]. The final concentration of the ssDNA bound to the surface of the gold nanoparticle was analysed by UV/Vis spectroscopy as described by McKenzie *et al.* [55].

Nanoparticle stability can be quantified by measuring the zeta potential of the colloidal system. If the particles in suspension have a large negative or positive zeta potential then they repel each other, reducing particle aggregation. However, if the particles have low zeta potential values, then there is no force to prevent particle flocculation. Generally, particles with zeta potentials more positive than +30 mV or more negative than −30 mV are considered stable [56]. The successful immobilization of DNA on the surface of the gold nanoparticles was ascertained by measuring the increase in the negative charge of the gold nanoparticles; the zeta potential of the functionalized gold nanoparticles was approximately −30 mV [46], [57].

### Transfection reagent-dependent HeLa cell death

In order to study the transfection efficiency of different transfection reagents, we transfected FITC-tagged ssDNA-functionalized nanoparticles into HeLa cells with a panel of three transfection reagents with different formulations, as follows. Matra (IBA) is a magnet-assisted nanoparticle-based transfection method for intracellular delivery of nucleic acid, while Lipofectamine 2000 (Invitrogen) is a cationic liposome-based reagent and GeneJuice (Novagen) consists of a non-toxic cellular protein and a small amount of a proprietary polyamine.

**Figure 1:**
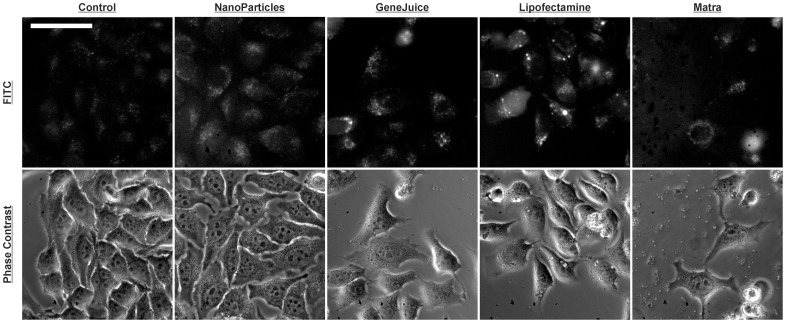
Fluoresence microscopy images showing localization of FITC-tagged hMT-IIa-specific sequence functionalized nanoparticles in live HeLa cells. HeLa cells were transfected with FITC-tagged hMT-IIa-specific sequence functionalized nanoparticles in the absence (NanoParticles column) or the presence of the transfection reagents Matra, Lipofectamine 2000 or GeneJuice. Control cells were treated with neither nanoparticles nor transfection reagents. The same signal intensity range was used for all FITC images. The scale bar represents 50 microns.

At first, and most importantly, we observed that the phase-contrast images of live HeLa cells showed that cells transfected with nanoparticles were largely adherent, with shapes that conformed to those of healthy HeLa cells for all the payloads considered, i.e. unfunctionalised nanoparticles, coated with sequences that were either not specifically targeted to a mammalian transcript or with sequences that targeted the human *MT-IIa* transcripts. While the transfection of nanoparticles with GeneJuice or Lipofectamine did not appear to affect the morphology of the HeLa cells, in contrast, very few HeLa cells treated with Matra survived the transfection protocol (Figure 1).

**Figure 2:**
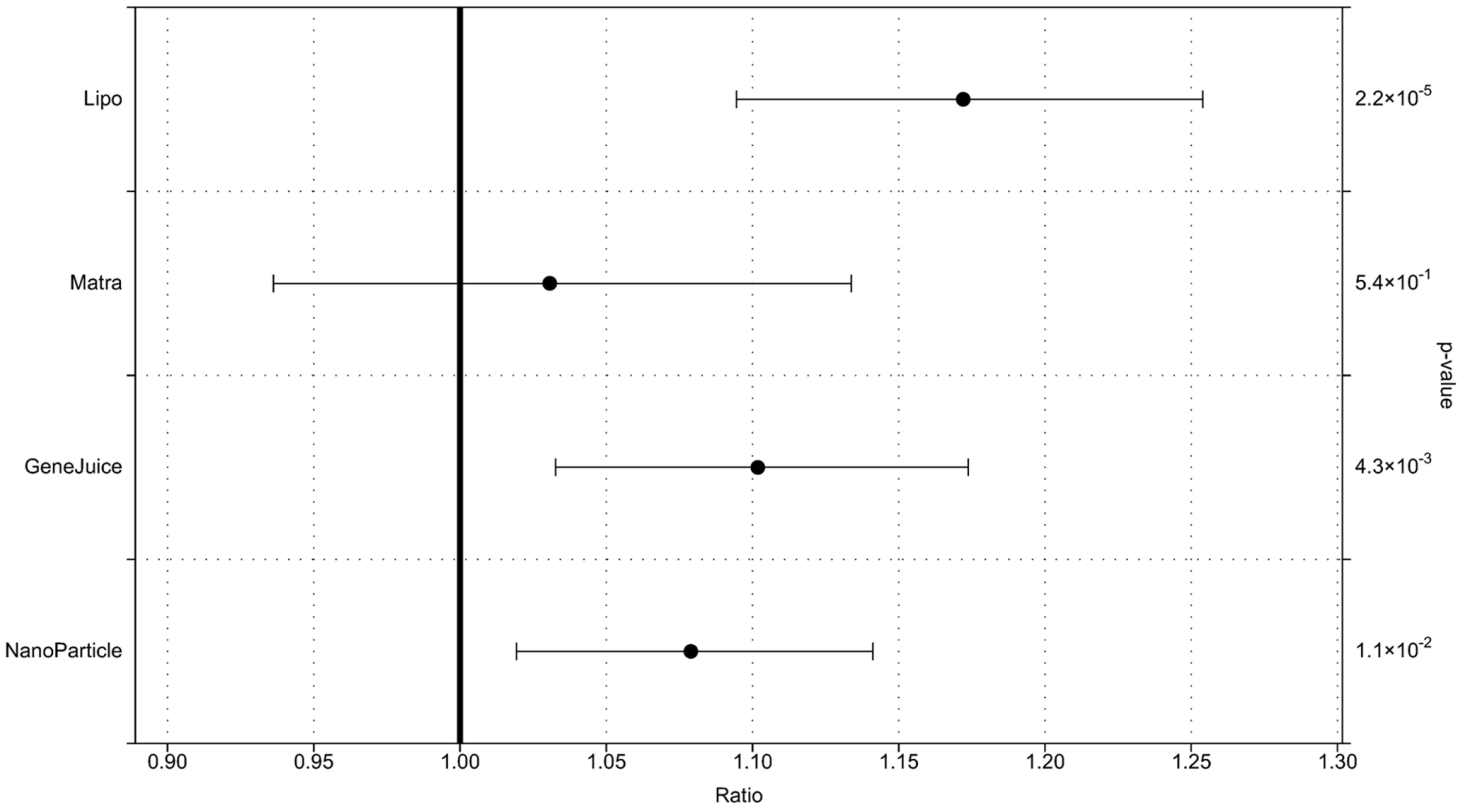
A statistical analysis of the level of FITC fluorescence in HeLa cells transfected with nanoparticles, in the presence of Lipofectamine, GeneJuice or Matra, compared to untransfected HeLa cells. HeLa cells were transfected with FITC-tagged hMT-IIa-specific sequence functionalized nanoparticles in the absence or the presence of transfection reagents. The FITC fluorescence in the cells was quantified as described in the methodology, and the levels of fluorescence in nanoparticle-transfected cells compared to those in untransfected cells. This multi-level statistical analysis takes into account local variations within the cell population. The bars represent the region where there is a 95% probability that the mean fluorescence increase lies within it. The dot represents the mean calculated effect size. Bars not crossing the 1 line show significant evidence for an effect following transfection. The p-value indicates the probability that there was no difference in expression levels between the control and treatment samples. Hence, a lower p-value indicates a greater likelihood that there was a difference between the transfected and the untransfected cells.

Fluorescence imaging of live HeLa cells transfected with functionalized gold nanoparticles (Figure 1) showed an increase in cellular fluorescence with respect to untransfected control cells (whose fluorescence signal results from native cellular autofluorescence). The measured whole-cell fluorescence emission of a number of individual cells for each transfection method was quantified and subsequent statistical analysis (Figure 2), using a two-level Bayesian ANOVA model, confirmed that the level of fluorescence from cells transfected with nanoparticles alone was significantly higher than for control cells.

**Figure 3:**
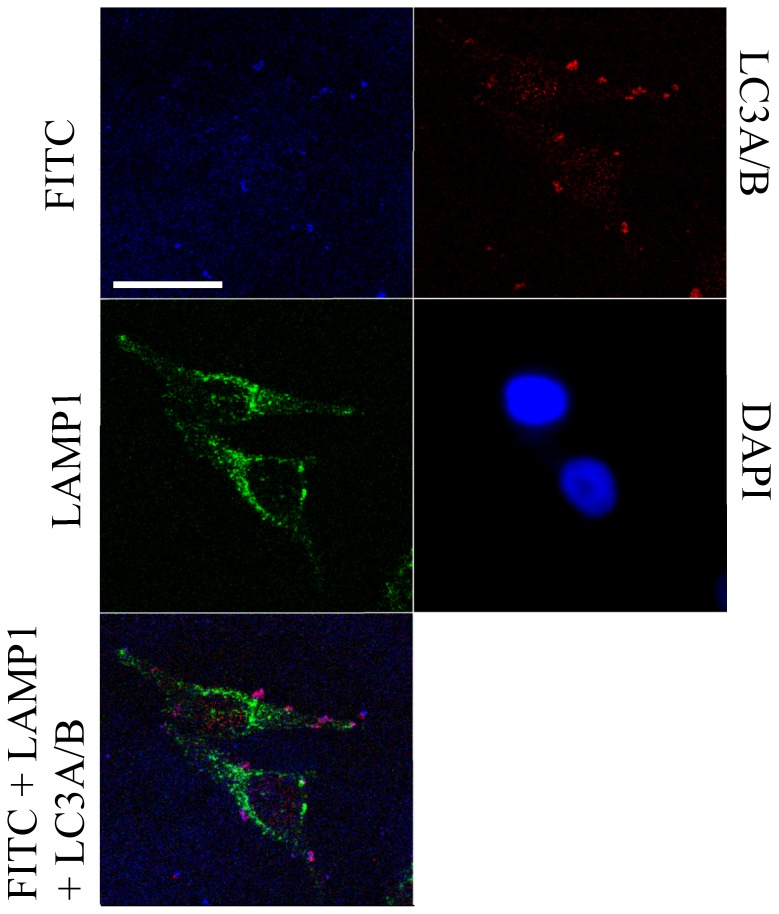
Confocal fluorescence microscopy images showing localization of FITC-tagged hMT-IIa-specific sequence functionalized nanoparticles in fixed HeLa cells. HeLa cells were transfected with FITC-tagged hMT-IIa-specific sequence functionalized nanoparticles in the presence of Lipofectamine. The overall FITC signal was reduced so that the level of fluorescence from the observed circular bodies was not saturating. The distribution of the LAMP1 protein extends through the majority of the cytoplasmic area thus provides an impression of the cell. In the overlayed image, the magenta regions indicate the presence of cellular bodies in which the FITC signal from the nanoparticles overlaps with the signal for the LC3A/B autophagosomal protein marker. The scale bar represents 25 microns and the images shown are all from a single z-plane (a 0.7 micron section).

Interestingly, the Lipofectamine- and, to a lesser extent, the GeneJuice-transfected live cells showed the presence of many strongly fluorescent circular bodies that were rarely seen in cells transfected with nanoparticles alone (Figure 1). To analyse these further, transfected HeLa cells were fixed and studied by fluorescent immunostaining for LC3A/B and LAMP1, protein markers for autophagosomes and lysosomes respectively. Fluorescence imaging of the LC3A/B and LAMP1 proteins showed that the signal intensity of these markers in untreated HeLa cells was comparable to that in HeLa cells that contained functionalized gold nanoparticles (Figure S2:647 and S2:546). When the fluorescence corresponding to LC3A/B protein (red) was overlayed with LAMP1 (green), there was no clear overlap between the autophagosomes and lysosomes indicating that these were distinct vesicular organelles (Figure S2:647+546, Figure 3).

As with the live cell studies, fixed cell fluorescence imaging of HeLa cells showed the presence of functionalized gold nanoparticles in both the cytoplasm and the nucleus, and increased signal was observed in cells transfected with GeneJuice and Lipofectamine (Figure S2:FITC). Bright circular bodies were again observed in the FITC channel in the transfected HeLa cells, particularly in the case of Lipofectamine-treated cells. Sometimes these bodies were observed inside the cells (ca. 20%, n = 10) but frequently (ca. 80%, n = 10) they were seen on the perimeter of the cell, or even outside the cell (Figure S2:FITC, Figure 3). Furthermore these circular bodies exhibited fluorescence in the LC3A/B channel (Figure S2:647, Figure 3) but not in the LAMP1 channel (Figure S2:546, Figure 3). Confocal microscopy confirmed that these LC3A/B containing vesicles co-localized with the FITC signal carried by the functionalized gold nanoparticles (Figure 3). Together the live cell and the immunofluorescence images suggest that these are autophagosomes containing functionalized gold nanoparticles that are either intracellular, are in the process of being extruded or have already been extruded from the cell.

The largest number of these bodies was observed when Lipofectamine was used as a transfection reagent; there were fewer bodies when HeLa cells were transfected with GeneJuice and such bodies were rare when no transfection reagent was used. This suggested two possibilities, that the lipid-based transfection reagent Lipofectamine enhanced exocytosis or that exocytosis was a factor of the increased transfection efficiency in the presence of Lipofectamine. Further experiments demonstrated that different concentrations of transfected functionalized gold nanoparticles did not affect the number of autophagosomes or extruded bodies and so the generation of these bodies is likely to be related to the transfection reagent. This highlights the importance of thorough validation of the reagents selected for use in nanoparticle-based biological studies as well as the inclusion of appropriate biological controls. For the knockdown studies, we selected GeneJuice as the transfection reagent of choice as it increased the endocytosis of the functionalized gold nanoparticles but did not introduce any detectable artefacts.

### Knockdown of MT gene expression

The majority of gene expression knockdown strategies use small interfering RNA (siRNA)-based techniques [58]–[60]. In contrast, most transfection studies of gold nanoparticles functionalized with nucleotides reported to date have been based on DNA, possibly due to its higher stability during the laboratory manipulations involved in preparing the reagents. Here, we compared the knockdown properties of ssDNA to the traditionally used siRNA and ssRNA. The siRNA sequences we used were comprised of sense and antisense RNA strands that have been annealed to form the siRNA-based inhibitor, whereas the ssRNA and ssDNA molecules consisted of single-stranded nucleic acid sequences that were complementary to the coding sequence.

**Figure 4:**
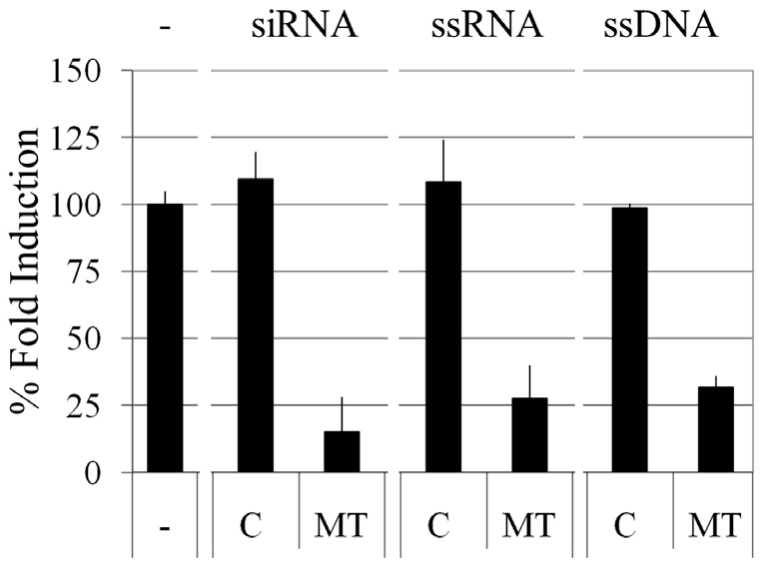
Reduction of hMTIIa gene expression in the presence of siRNA, ssRNA and ssDNA. HeLa cells were untransfected (−), transfected with 5 nM control (C) or 5 nM hMTIIa (MT)-specific siRNA, ssRNA or ssDNA sequences using GeneJuice (Novagen). All samples were induced with 12.5 µM CdCl_2_. The level of hMTIIa gene expression (normalized to *B2M*) in untransfected and induced HeLa cells was defined as 100% and all other fold inductions were expressed relative to this. The error bars were calculated as 1 standard error of the mean each way.

A system was designed for the detection and quantification of *hMT-IIa* gene expression (Text S1, Figure S3 and Figure S4). HeLa cells were transfected with control or hMT-IIa-specific nucleotide sequences and the levels of hMT-IIa transcripts following CdCl_2_ treatment were compared. When hMT-IIa-specific siRNA, ssRNA or ssDNA sequences were transfected into HeLa cells, a marked reduction in the levels of hMT-IIa mRNA transcripts was observed (Figure 4) compared to random control sequences, where no suppression was observed. At these concentrations (5 nM), the levels of the hMT-IIa transcripts in HeLa cells transfected with siRNA, ssRNA and ssDNA were comparable between the different oligonucleotide types (Figure 4), and similar knockdown efficiency was observed with all three types of inhibitory sequences (from 100% in the controls to 15±13% for siRNA, 28±12% for ssRNA and 32±4% for ssDNA). The siRNA and ssRNA-based knockdown of hMT-IIa transcripts showed greater variation between the biological replicates than when ssDNA was used as the targeting nucleotide (Figure 4); coefficient of variation (CV) for siRNA and ssRNA experiments were 0.5 and 0.3 respectively, whereas ssDNA showed a CV of 0.08. This may reflect the greater stability of ssDNA compared to siRNA and ssRNA.

**Figure 5:**
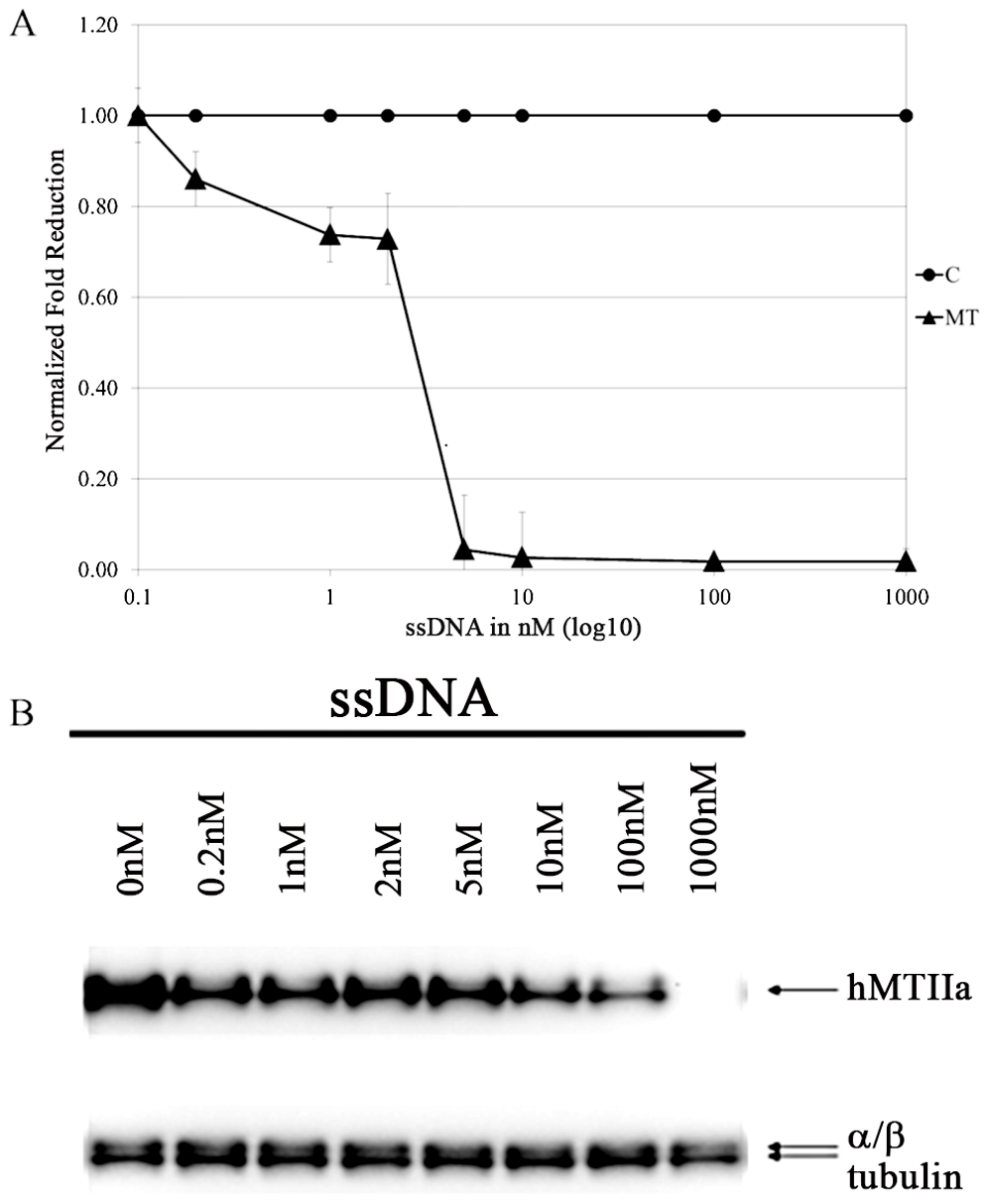
Levels of hMT-IIa in HeLa cells transfected with increasing concentrations of hMT-IIa or control ssDNA. Transfections were carried out with GeneJuice (Novagen). (A) Levels of hMT-IIa mRNA. All samples were induced samples with 12.5 µM CdCl_2_. Total hMT-IIa activity relative to *B2M* in cells transfected with varying levels of ssDNA was normalized to hMT-IIa-*B2M* expression in cells transfected with 0 nM ssDNA giving us a value for hMT-IIa activity in the presence of x nM ssDNA. The levels of hMT-IIa transcript in cells transfected with control ssDNA (C) was set to 1.0. The level of hMT-IIa gene expression (MT) in cells transfected with hMT-IIa ssDNA was compared to the levels of hMT-IIa in cells transfected with control ssDNA at each concentration providing a measure of the reduction in hMT-IIa activity in hMT-IIa ssDNA transfected compared to those transfected with control ssDNA. The error bars were calculated as 1 standard error of the mean each way. (B) Levels of hMT-IIa protein. All samples were induced with 12.5 µM CdCl_2_. HeLa cells were transfected with hMT-IIa-specific ssDNA (ssDNA). Proteins extracted from HeLa cells were analyzed using Western blots and hMT-IIa-specific antibodies. α/β tubulin was used as a loading control. Quantification of the levels of hMT-IIA protein relative to α/β tubulin is shown in Table S2B.

We then defined the lowest effective concentration of hMT-IIa-specific ssDNA required for the reduction of the hMT-IIa mRNA and protein levels (Figure 5A and 5B). For concentrations of hMT-IIa ssDNA between 0.2 to 2 nM, there was a noticeable decrease in transcript levels (0.86 to 0.73 normalized fold reduction compared to cells transfected with control sequences); while at concentrations >5 nM, the levels of hMT-IIa transcripts in the HeLa cells decreased to less than 5% of the initial hMT-IIa transcript levels (Figure 5A). This reduction in the levels of the hMT-IIa transcripts was also reflected at the protein level. As the concentration of hMT-IIa ssDNA transfected into HeLa cells increased, the levels of the hMT-IIA protein detected by Western blot analysis decreased (Figure 5B, Table S2B). At a concentration of 1000 nM ssDNA, the hMT-IIA protein was almost undetectable (Figure 5B).

**Figure 6:**
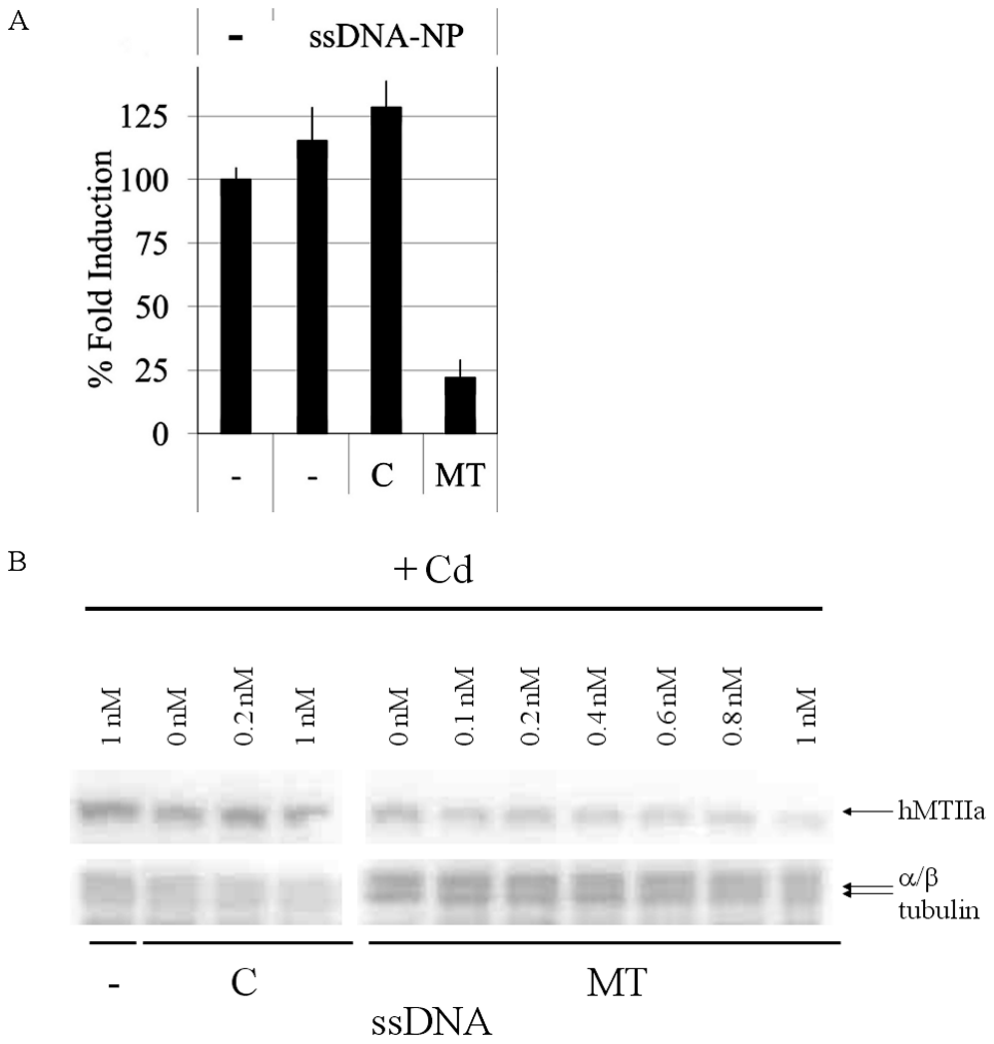
Levels of hMTIIa in HeLa cells transfected with gold nanoparticles. Transfections were carried out with GeneJuice (Novagen). (A) Levels of hMT-IIa mRNA. HeLa cells were transfected with 5 nM unfunctionalized (−), 5 nM control (C) or 5 nM hMTIIa (MT)-specific ssDNA functionalized gold nanoparticles. Samples were treated with 12.5 µM CdCl_2_. The level of hMTIIa gene expression (normalized to *B2M*) in untransfected and induced HeLa cells was defined as 100% and all other fold inductions were expressed relative to this. The error bars were calculated as 1 standard error of the mean each way. (B) Levels of hMT-IIa protein. Induced samples were treated with 12.5 µM CdCl_2_ (+Cd). HeLa cells were transfected with unfunctionalized (−), control (C) or hMT-IIa (MT) ssDNA-functionalized nanoparticles. Proteins extracted from HeLa cells were analyzed using Western blots and hMT-IIa-specific antibodies. α/β tubulin was used as a loading control. Quantification of the levels of hMT-IIA protein relative to α/β tubulin is shown in Table S2C.

The ssDNA-based reduction in the level of hMT-IIa mRNA levels in the cells also occurred when the hMT-IIa-specific ssDNA sequence functionalized nanoparticles were used, but not with control sequences or bare gold nanoparticles. In this case, gold nanoparticles functionalized with the same concentration of ssDNA as that used above (5 nM) were transfected into HeLa cells. There was no significant difference between the levels of hMT-IIa mRNA transcripts between immobilized and free oligonucleotide (Figure 6A vs. Figure 4), indicating that the attachment of the ssDNA sequences to the gold nanoparticles did not affect their ability to knock-down hMT-IIa mRNA levels. The hMT-IIa ssDNA functionalized dependent decrease in hMT-IIa mRNA transcripts was detected for 72 h (Figure S5). In addition, the reduction in the level of the hMT-IIa transcript was greater when GeneJuice was used as a transfection reagent, to assist internalization of the ssDNA functionalized nanoparticles, than when no tranfection reagent was present (Figure S6). A reduction in hMT-IIA protein was also detected when nanoparticles functionalized with hMT-IIa-specific ssDNA (ssDNA-NP) were transfected into cells, but not when unmodified or control sequence functionalized nanoparticles were used (Figure 6B, Table S2C). These results demonstrate that the reduction in hMT-IIa mRNA transcripts was also reflected at the level of hMT-IIA protein.

Quantification of the Western blot images (Table S2B) showed that the hMT-IIA protein was reduced to 50% of the initial levels after transfection with 0.2 nM hMTIIa-specific ssDNA. At concentrations of 1000 nM, hMTIIA protein was knocked down to undetectable levels (Table S2B). To determine if ssDNA functionalized gold nanoparticles behaved in a similar manner to free ssDNA, DNA functionalized gold nanoparticles were transfected into HeLa cells at concentrations between 0 nM and 1 nM; which were non-toxic to the cells and were equivalent to the levels of free ssDNA that reduced the level of the hMTIIA protein to 31% of the initial levels (Table S2B). When hMTIIa-specific sequences were used to functionalize gold nanoparticles, the level of hMTIIA protein expression in cells transfected with 1 nM nanoparticles was reduced to 27% of that measured in HeLa cells transfected with control-sequence functionalized nanoparticles or unfunctionalized nanoparticles (Table S2C). This data shows that the ssDNA-functionalized nanoparticles and free ssDNA elicited similar concentration-dependent responses in CdCl_2_ treated HeLa cells.

It is interesting to note that when the mRNA transcripts were quantified, at concentrations of ssDNA >5 nM, the levels of hMT-IIa were undetectable. In contrast, at the same ssDNA concentration, hMT-IIA protein levels were still detected and full knockdown was observed only at 1000 nM ssDNA. It is possible that the antibody directed against the hMT-IIa protein detects hMT isoforms other than hMT-IIa; and this non-specific activity may be exacerbated by the relatively long half-life of the MT proteins in the cell [61].

Each gold nanoparticle was functionalized with approximately 200 oligonucleotide strands [62] thus each particle has a finite binding capacity for cellular *hMTIIa* gene transcript. In this knockdown of gene expression study, we used a maximum of 1 nM gold nanoparticles in a transfection to avoid cytotoxicity. It is possible that at these concentrations, not all the cellular *hMTIIa* gene transcript would be bound to the transfected ssDNA oligonucleotides and so some cellular transcripts may remain in solution. To determine whether this was the case, total RNA isolated from HeLa cells that had been transfected with unfunctionalized, control or hMTIIA-specific sequence functionalized gold nanoparticles and incubated for 16 h was analyzed for levels of the hMTIIa transcript by qPCR. HeLa cells transfected with unfunctionalized or control-sequence functionalized nanopartices resulted in no reduction in the level of the hMTIIa transcript whereas HeLa cells transfected with hMT-IIa sequence functionalized nanoparticles resulted in a reduction in the hMTIIa transcript to 11.1% (±9.8%) of the initial levels (Table 1). This indicates that there was little free hMT-IIa transcript remaining in the cell after incubation with hMTIIa sequence functionalized nanoparticles.

**Table 1:** Quantification of the levels of hMT-IIa transcript in HeLa cells after treatment with ssDNA-functionalized nanoparticles.

Gold nanoparticles	% hMT-IIA
-	100±6.0
C	114.5±10.4
MT	11.1±9.8

Induced samples were treated with 12.5 µM CdCl_2_ (+Cd). HeLa cells were transfected with 5 nM unfunctionalized (−), 5 nM control (C) or 5 nM hMTIIa (MT)-specific ssDNA functionalized gold nanoparticles. The level of hMTIIa gene expression (normalized to *B2M*) in HeLa cells transfected with unfunctionalized (−)gold nanoparticles was normalized to 100% and all other fold inductions were expressed relative to this. The error bars were calculated as 1 standard error of the mean each way and represented as a percentage of the activity.

## Discussion

For this study, we required a robust, rapidly growing, cell line that could be reproducibly transfected [63] that was able to express MT-IIA (and the expression of this gene needed to be inducible [39], [64]). Based on the requirements of this study, we selected the HeLa cell line. The goal of this knockdown study was the efficient quantifiable knockdown of target expression in the context of the biological targeting event. The methods available to measure target knockdown include Western blot analysis of the target protein (which is semi-quantitative and dependent on the availability of sensitive and specific antibodies) and qPCR (which is quantitative and any gene of interest can be analysed after the design of appropriate primers). The analysis of the levels of target mRNA is robust and allows for reproducible biological knockdown studies. It does not however measure the effect of targeted knockdown. Based on our observations, we propose that the measurement of both mRNA and protein are required for a true reflection of specific target knockdown in the cell.

The efficiency with which nanoparticles enter the cell has been a major focus of research. While the nanoparticles themselves enter the cell and the nucleus, it is clear that the use of transfection reagents improves the efficiency of this process. We observed that the use endocytosis-based transfection reagents (Lipofectamine 2000 and GeneJuice) resulted in an increased efficiency of nanoparticle transfection. In contrast, the use of the nanoparticle-based transfection reagent Matra under these conditions resulted in cell death. As the efficiency of target delivery directly influences knockdown efficiency, it is important both to better understand how these transfection reagents enhance particle delivery and to develop reagents for the targeted delivery of nanoparticles into the cells. The increased efficiency of targeted ssDNA delivery directly to the nucleus means that lower concentrations of inhibitory sequences need to be used to elicit a biological effect. This is an advantage when dealing with potentially toxic sequences or genes that are expressed at higher levels.

In this report, we defined the ssDNA-functionalized gold nanoparticles by the concentration of DNA attached to the nanoparticles. Based on the data that we collected, we observed that that the ssDNA behaved comparably at similar concentrations whether it was free or functionalized onto gold nanoparticles. The advantages of using nanoparticles are numerous; including the fact that the functionalized nanoparticles entered HeLa cells both in the presence and the absence of transfection reagents which could negate the need for transfection reagents. This is an important factor when considering *in vivo* experiments. In contrast, the use of transfection reagents enhanced transfection efficiency, allowing the use of lower concentrations of functionalized nanoparticles in biological experiments, with direct effects on the cost of the reagents. The nanoparticles provided a structure onto which to bind the ssDNA sequences. This allows the same ssDNA-functionalized nanoparticles to be used in both knockdown and fluorescence microscopy studies, providing directly comparable biological and visual data. Finally, it has been shown previously that antibody-functionalized nanoparticles can be used to target particular cell types; hence attaching both targeting antibodies and inhibitory ssDNA to these nanoparticles is an attractive option and the next step.

## Conclusions

In this article, we demonstrate (a) that the validation of experimental reagents and the inclusion of appropriate controls is essential to avoid the collection and over-interpretation of artifacts in the data and (b) that ssDNA inhibitory sequences are as effective as ssRNA and siRNA inhibitory sequences at targeting knockdown of a specific gene target, both at the mRNA transcript and the protein level.

## Supporting Information

Figure S1 **Measuring the size of a colloidal suspension of the FITC-labelled ssDNA-functionalized gold nanoparticles by dynamic light scattering.** Three particle number-based size distribution measurements were performed on a suspension of nanoparticles in dH_2_O. Averaging the three measurements (each of which is the output of 20 runs) gave a peak diameter of 31 nm.
(TIF)

Figure S2 **Fluorescence microscopy images showing localization of FITC-tagged hMT-IIa-specific sequence functionalized nanoparticles in fixed HeLa cells.** (A) HeLa cells were transfected with FITC-tagged hMT-IIa-specific sequence functionalized nanoparticles in the absence or the presence of transfection reagents Lipofectamine or GeneJuice. The control samples include HeLa cells probed for the lysosomal marker with anti-LAMP1 and anti-Alexa546 and for the autophagosomal marker with anti-LC3A/B and anti-Alexa647. Finally, control samples represent HeLa cells that were probed with only a secondary antibody. Comparable results were obtained for anti-Alexa546 alone and anti-Alexa647 alone, and the data for anti-Alexa647 is shown here. A fixed signal intensity range for each spectral channel was used for fluorescence images. Circles have been drawn around structures with signal in the anti-FITC and the anti-Alexa647 channels. The scale bar represents 50 microns. (B) Images for HeLa cells transfected with FITC-tagged hMT-IIa-specific sequence functionalized nanoparticles in the presence of Lipofectamine (from Figure S2A) have been enlarged. Circles show structures with signal in the anti-FITC and the anti-Alexa647 channels; the scale bar represents 50 microns.
(TIFF)

Figure S3 **Induction of** ***hMTIIa*** **gene expression in increasing CdCl_2_ concentrations in HeLa cells.** Expression of *hMTIIa* was normalized to that of B2M, a stably-expressed chromosomal gene for beta-2-microglobulin. The level of hMTIIa gene expression in uninduced HeLa cells was defined as the basal level of expression (1 arbitrary unit) and all other fold inductions were expressed relative to this. The error bars were calculated as 1 standard error of the mean each way.
(TIF)

Figure S4 **Induction of hMTIIa expression in the presence of Cd.** Untransfected HeLa cells were treated with 12.5 µM CdCl_2_ (+Cd). (A) The level of hMTIIa gene expression (normalized to *B2M*) in induced HeLa cells was defined as 100% and the fold induction of the uninduced cells was expressed relative to this. The error bars were calculated as 1 standard error of the mean each way. (B) The level of hMT-IIa protein in induced HeLa cells. Proteins extracted from HeLa cells were analyzed using Western blots and hMT-IIa-specific antibodies. α/β tubulin was used as a loading control. Quantification of the levels of hMT-IIA protein relative to α/β tubulin is shown in Table S2A.
(TIF)

Figure S5 **Reduction of hMTIIa gene expression in the presence of Cd over 72 hours.** HeLa cells were transfected with 5 nM control (C) or 5 nM hMT-IIa (MT) specific ssDNA sequence functionalized nanoparticles. Transfections were carried out with GeneJuice (Novagen). Total hMT-IIa activity relative to *B2M* in cells transfected with 5 nM hMT-IIa (MT) specific ssDNA sequence functionalized nanoparticles was normalized to hMT-IIa-*B2M* expression in cells transfected with control sequence functionalized nanoparticles. The levels of hMT-IIa transcript in cells at Time = 0 h was set to 100%. The level of hMT-IIa gene expression (MT) in cells transfected with hMT-IIa ssDNA functionalized gold nanoparticles was compared to the levels of hMT-IIa in cells transfected with control ssDNA functionalized gold nanoparticles at each time point providing a measure of the reduction in hMT-IIa activity in cells transfected with hMT-IIa (MT) compared to those transfected with control (C) ssDNA functionalized nanoparticles. The error bars were calculated as 1 standard error of the mean each way.
(TIFF)

Figure S6 **The effect of the transfection reagent GeneJuice on the knockdown of hMTIIa gene expression in the presence of Cd.** HeLa cells were transfected with 5 nM control (C) or 5 nM hMT-IIa (MT) specific ssDNA sequence functionalized nanoparticles. Total hMT-IIa activity relative to *B2M* in cells transfected with 5 nM hMT-IIa (MT) specific ssDNA sequence functionalized nanoparticles was normalized to hMT-IIa-*B2M* expression in cells transfected with control sequence functionalized nanoparticles. The levels of hMT-IIa transcript in cells transfected with control (C) sequence functionalized nanoparticles was set to 100%. The level of hMT-IIa gene expression (MT) in cells transfected with hMT-IIa ssDNA functionalized gold nanoparticles was compared to the levels of hMT-IIa in cells transfected with control ssDNA functionalized gold nanoparticles providing a measure of the reduction in hMT-IIa activity in cells transfected with hMT-IIa (MT) compared to those transfected with control (C) ssDNA functionalized nanoparticles. The error bars were calculated as 1 standard error of the mean each way.
(TIFF)

Table S1 **Primers used in this study.**
(DOCX)

Table S2 **Quantification of the reduction in hMT-IIA protein in HeLa cells treated with ssDNA or ssDNA-functionalized nanoparticles in the Western blots.** Induced samples were treated with 12.5 µM CdCl_2_ (+Cd).
(DOC)

Text S1 **Design and optimization of a system for MT gene expression analysis.**
(DOCX)
