# Supplementary figures and images for: Quantification of Functionalised Gold Nanoparticle-Targeted Knockdown of Gene Expression in HeLa Cells
Source: PLoS One. 2014 Jun 13;9(6):e99458. doi: 10.1371/journal.pone.0099458 (PMC4057226; doi:10.1371/journal.pone.0099458)

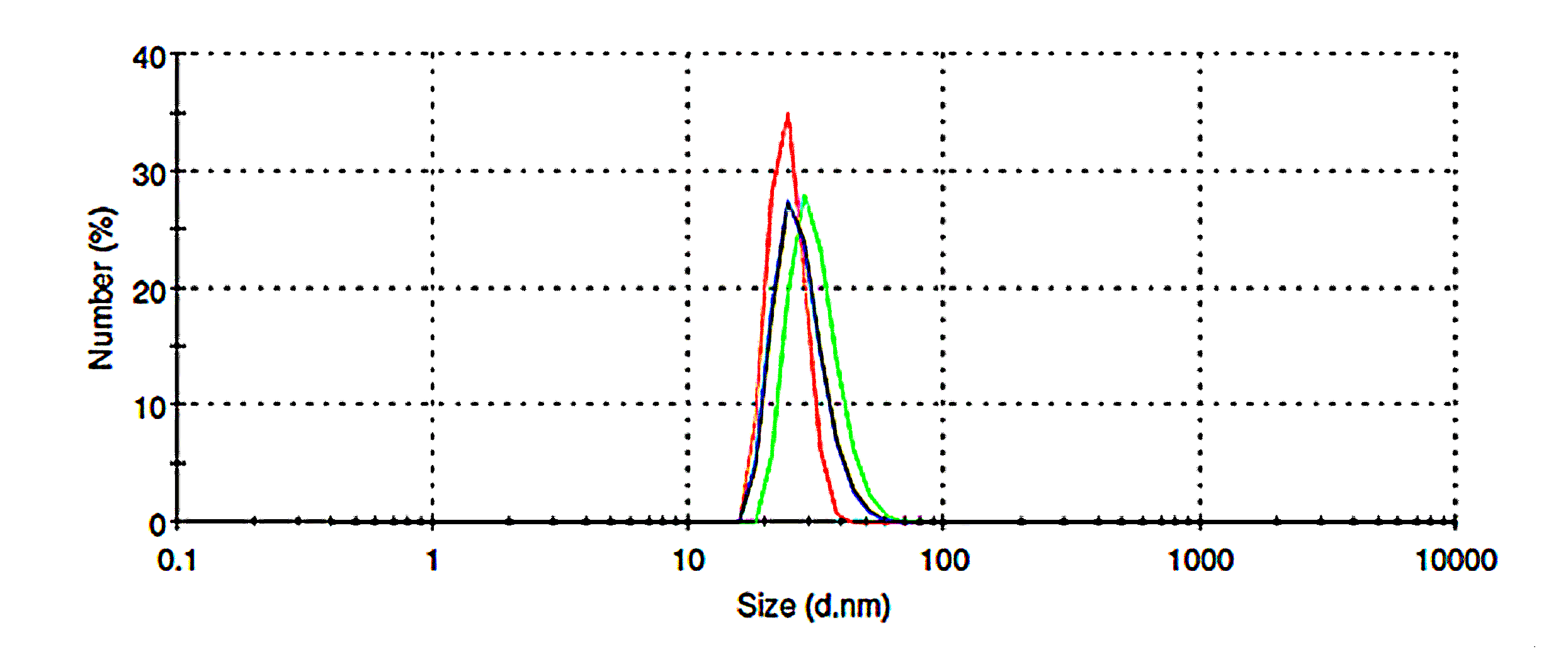

Supplement: Figure S1 — Measuring the size of a colloidal suspension of the FITC-labelled ssDNA-functionalized gold nanoparticles by dynamic light scattering. Three particle number-based size distribution measurements were performed on a suspension of nanoparticles in dH2O. Averaging the three measurements (each of which is the output of 20 runs) gave a peak diameter of 31 nm. (TIF) [file pone.0099458.s001.tif]

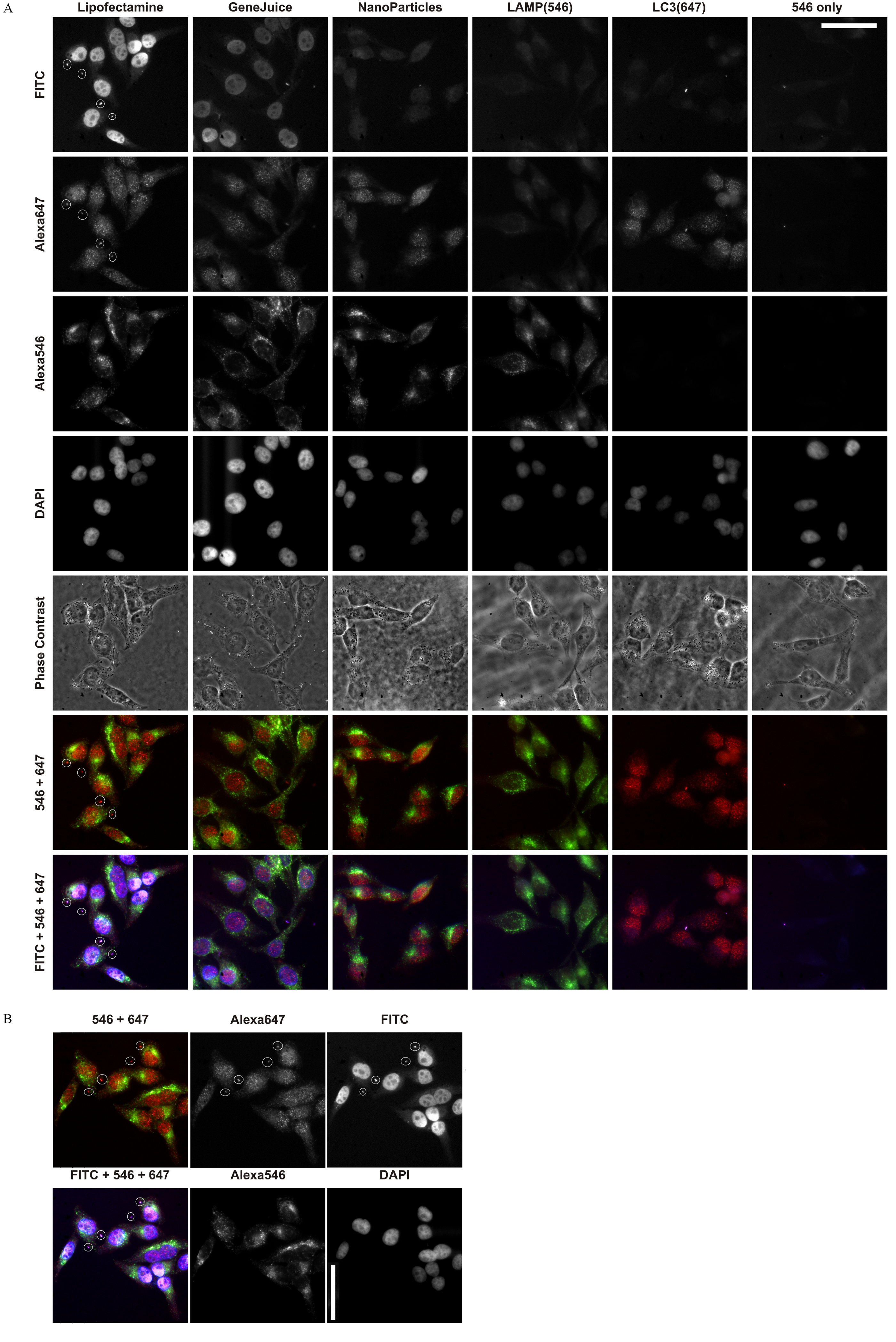

Supplement: Figure S2 — Fluorescence microscopy images showing localization of FITC-tagged hMT-IIa-specific sequence functionalized nanoparticles in fixed HeLa cells. (A) HeLa cells were transfected with FITC-tagged hMT-IIa-specific sequence functionalized nanoparticles in the absence or the presence of transfection reagents Lipofectamine or GeneJuice. The control samples include HeLa cells probed for the lysosomal marker with anti-LAMP1 and anti-Alexa546 and for the autophagosomal marker with anti-LC3A/B and anti-Alexa647. Finally, control samples represent HeLa cells that were probed with only a secondary antibody. Comparable results were obtained for anti-Alexa546 alone and anti-Alexa647 alone, and the data for anti-Alexa647 is shown here. A fixed signal intensity range for each spectral channel was used for fluorescence images. Circles have been drawn around structures with signal in the anti-FITC and the anti-Alexa647 channels. The scale bar represents 50 microns. (B) Images for HeLa cells transfected with FITC-tagged hMT-IIa-specific sequence functionalized nanoparticles in the presence of Lipofectamine (from Figure S2A) have been enlarged. Circles show structures with signal in the anti-FITC and the anti-Alexa647 channels; the scale bar represents 50 microns. (TIFF) [file pone.0099458.s002.tif]

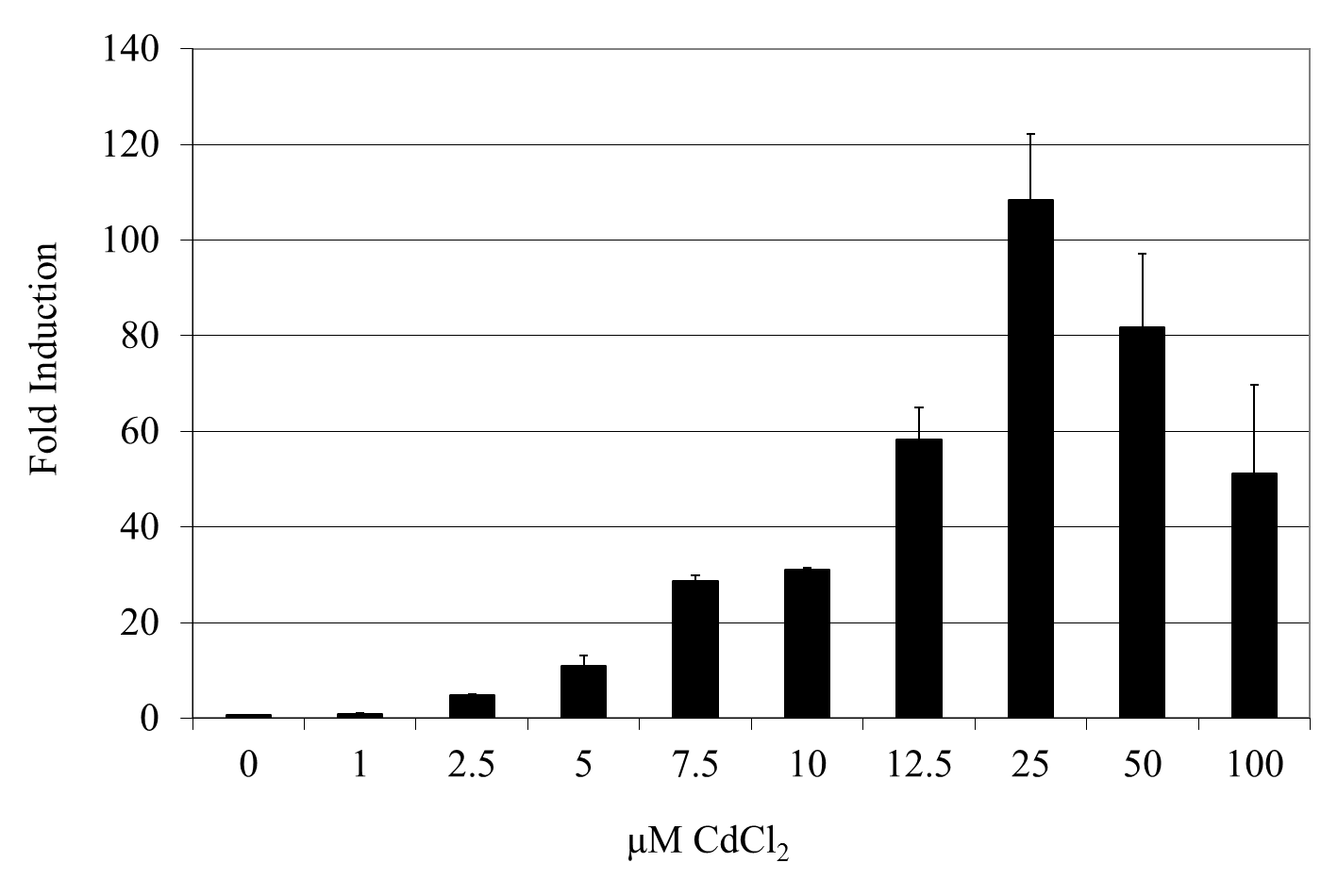

Supplement: Figure S3 — Induction of hMTIIa gene expression in increasing CdCl2 concentrations in HeLa cells. Expression of hMTIIa was normalized to that of B2M, a stably-expressed chromosomal gene for beta-2-microglobulin. The level of hMTIIa gene expression in uninduced HeLa cells was defined as the basal level of expression (1 arbitrary unit) and all other fold inductions were expressed relative to this. The error bars were calculated as 1 standard error of the mean each way. (TIF) [file pone.0099458.s003.tif]

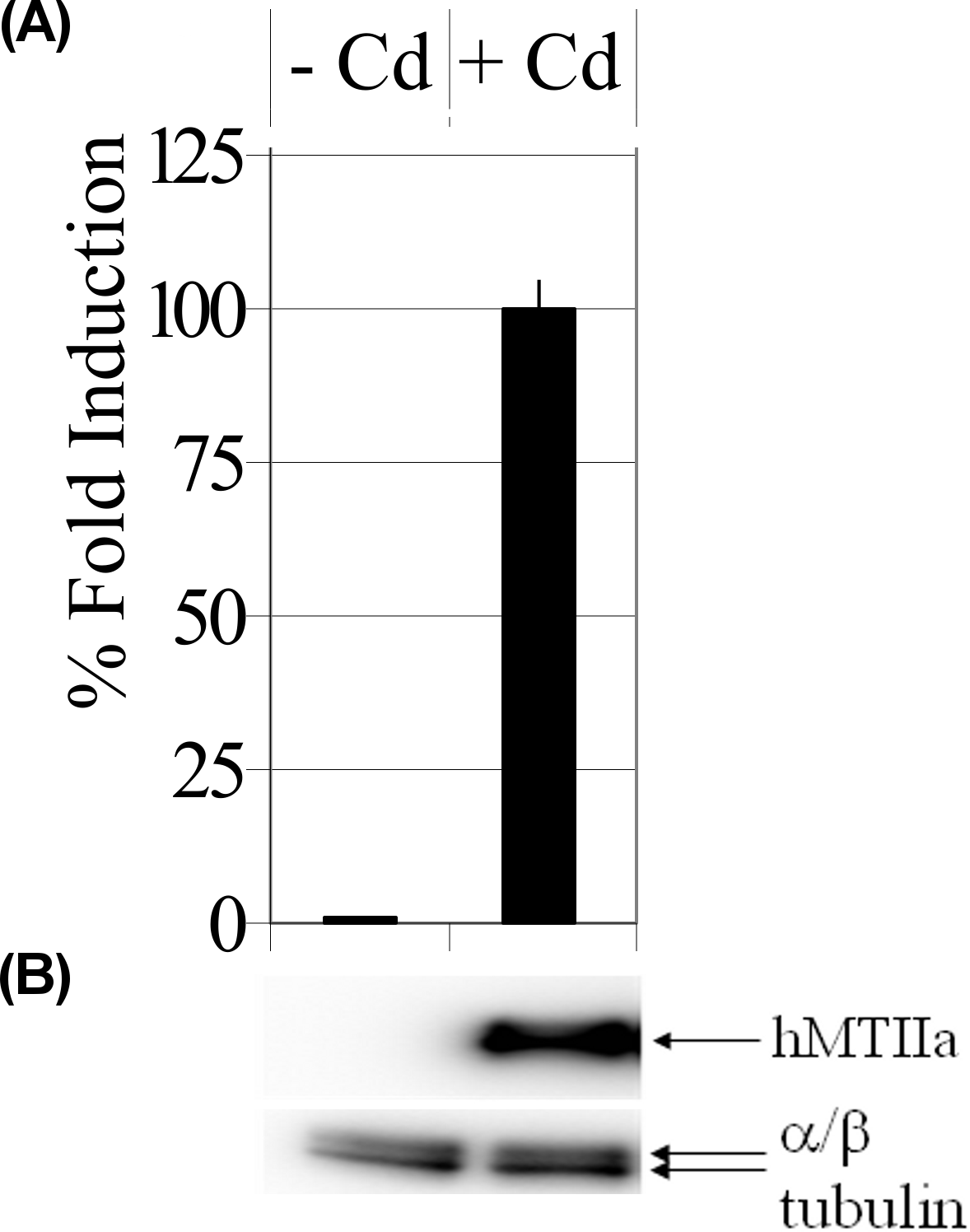

Supplement: Figure S4 — Induction of hMTIIa expression in the presence of Cd. Untransfected HeLa cells were treated with 12.5 µM CdCl2 (+Cd). (A) The level of hMTIIa gene expression (normalized to B2M) in induced HeLa cells was defined as 100% and the fold induction of the uninduced cells was expressed relative to this. The error bars were calculated as 1 standard error of the mean each way. (B) The level of hMT-IIa protein in induced HeLa cells. Proteins extracted from HeLa cells were analyzed using Western blots and hMT-IIa-specific antibodies. α/β tubulin was used as a loading control. Quantification of the levels of hMT-IIA protein relative to α/β tubulin is shown in Table S2A. (TIF) [file pone.0099458.s004.tif]

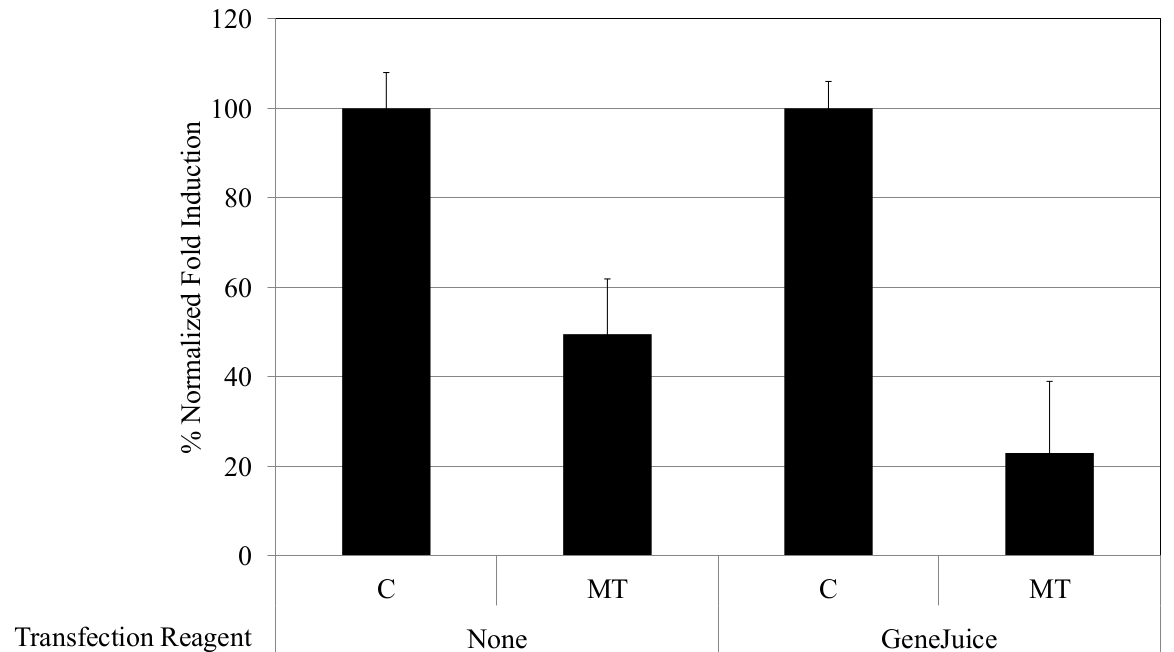

Supplement: Figure S6 — The effect of the transfection reagent GeneJuice on the knockdown of hMTIIa gene expression in the presence of Cd. HeLa cells were transfected with 5 nM control (C) or 5 nM hMT-IIa (MT) specific ssDNA sequence functionalized nanoparticles. Total hMT-IIa activity relative to B2M in cells transfected with 5 nM hMT-IIa (MT) specific ssDNA sequence functionalized nanoparticles was normalized to hMT-IIa-B2M expression in cells transfected with control sequence functionalized nanoparticles. The levels of hMT-IIa transcript in cells transfected with control (C) sequence functionalized nanoparticles was set to 100%. The level of hMT-IIa gene expression (MT) in cells transfected with hMT-IIa ssDNA functionalized gold nanoparticles was compared to the levels of hMT-IIa in cells transfected with control ssDNA functionalized gold nanoparticles providing a measure of the reduction in hMT-IIa activity in cells transfected with hMT-IIa (MT) compared to those transfected with control (C) ssDNA functionalized nanoparticles. The error bars were calculated as 1 standard error of the mean each way. (TIFF) [file pone.0099458.s006.tif]
