# Supplementary material for: Quantification of Functionalised Gold Nanoparticle-Targeted Knockdown of Gene Expression in HeLa Cells
Source: PLoS One. 2014 Jun 13;9(6):e99458. doi: 10.1371/journal.pone.0099458 (PMC4057226; doi:10.1371/journal.pone.0099458)
Supplement: Table S1 — Primers used in this study. (DOCX) [file pone.0099458.s007.docx]

**Table S1: Primers used in this study**

|  | Probe | Application | Sequence |
| --- | --- | --- | --- |
| hMTIIa | prMJ313 | qPCR | gtgggctgtgccaagtgt |
|  | prMJ314 | qPCR | FAM-cgacttccacaaacctggat-BHQ1 |
|  | prMJ315 | qPCR | atagcaaacggtcacggtca |
| B2M | prMJ348 | qPCR | TCTCTGCTCCCCACCTCTAA |
|  | prMJ349 | qPCR | FAM-CCAGCCCTCCTAGAGCTACC-BHQ1 |
|  | prMJ350 | qPCR | ATCTGAGCAGGTTGCTCCAC |
| hMTIIa-DNA | prMJ514 | DNA Silencer | TTTGTGGAAGTCGCGTTCTTT |
| Control-DNA | prMJ516 | DNA Silencer | TTACGTCGTCGCGTCGTTATT |
| hMTIIa-RNA | prMJ514 | RNA Silencer | UUUGUGGAAGUCGCGUUCUUU |
| Control-RNA | prMJ516 | RNA Silencer | UUACGUCGUCGCGUCGUUAUU |
| hMTIIa-siRNA | prMJ514 | siRNA Silencer | AGAACGCGACUUCCACAAAtt  UUUGUGGAAGUCGCGUUCUtt |
| Control-siRNA | prMJ516 | siRNA Silencer | UAACGACGCGACGACGUAATT  UUACGUCGUCGCGUCGUUATT |
