# Supplementary material for: Quantification of Functionalised Gold Nanoparticle-Targeted Knockdown of Gene Expression in HeLa Cells
Source: PLoS One. 2014 Jun 13;9(6):e99458. doi: 10.1371/journal.pone.0099458 (PMC4057226; doi:10.1371/journal.pone.0099458)
Supplement: Table S2 — Quantification of the reduction in hMT-IIA protein in HeLa cells treated with ssDNA or ssDNA-functionalized nanoparticles in the Western blots. Induced samples were treated with 12.5 µM CdCl2 (+Cd). (DOC) [file pone.0099458.s008.doc]

**Table S2: Quantification of the reduction in hMT-IIA protein in HeLa cells treated with ssDNA or ssDNA-functionalized nanoparticles in the Western blots.** Induced samples were treated with 12.5 μM CdCl_2_ (+ Cd).

(A) The level of hMT-IIA protein in HeLa cells that were untreated or treated with CdCl_2_ (Figure S4B). The level of hMT-IIA protein in HeLa cells treated with CdCl_2_ was normalized to 100% and all other values are expressed relative to this value.

| **Sample** | **% hMT-IIA** |  | | |
| --- | --- | --- | --- | --- |
| HeLa cells –Cd | 8% |  | | |
| HeLa cells +Cd | 100% |  | | |
|  | | | |  |
| (B) The level of hMT-IIA protein in HeLa cells transfected with an increasing concentration of ssDNA (Figure 5B). The level of hMT-IIA protein in HeLa cells transfected with 0 nM ssDNA was normalized to 100% and all other values are expressed relative to this value. | | | |  |
| **ssDNA concentration (nM)** | **% hMT-IIA** | |  | |
| 0 | 100% | | 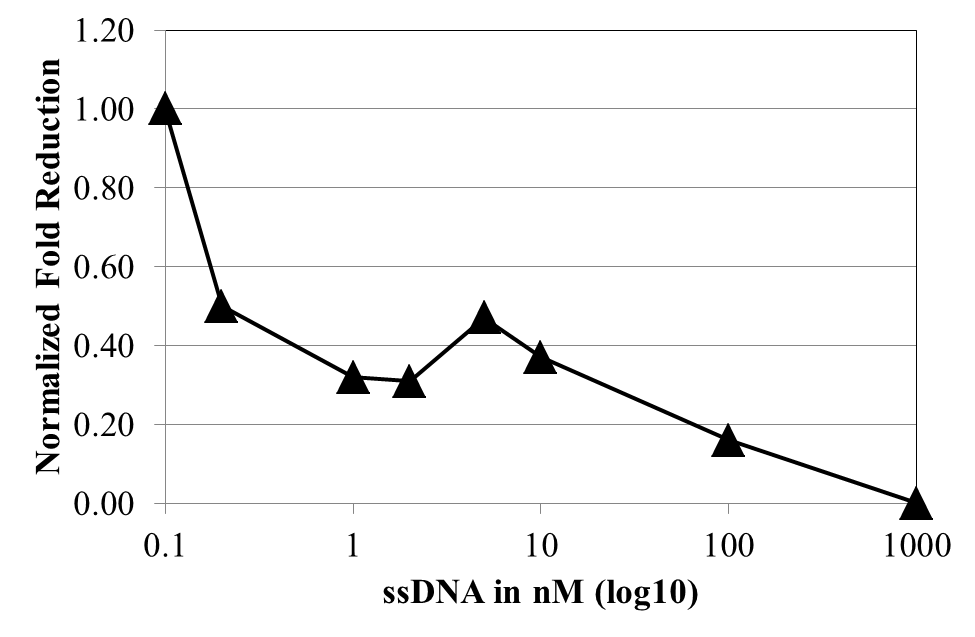 | |
| 0.2 | 50% | |
| 1 | 31% | |
| 2 | 31% | |
| 5 | 47% | |
| 10 | 37% | |
| 100 | 16% | |
| 1000 | 0% | |
|  | | | |  |

| (C) The level of hMT-IIA protein in HeLa cells transfected with control and hMT-IIa sequence functionalized gold nanoparticles (Figure 6B). The level of hMT-IIA protein in HeLa cells transfected with 0 nM control ssDNA-NPs was normalized to 100% and all other values are expressed relative to this value. | | | |  |
| --- | --- | --- | --- | --- |
| **NPs** | | **% hMT-IIA** |  | |
| *-* ssDNA | 1 nM | 116% |  | |
| C ssDNA | 0 nM | 100% |  | |
|  | 0.2 nM | 97% |  | |
|  | 1 nM | 98% |  | |
| MT ssDNA | 0 nM | 97% |  | |
|  | 0.1 nM | 65 % |  | |
|  | 0.2 nM | 53% |  | |
|  | 0.4 nM | 48% |  | |
|  | 0.6 nM | 40% |  | |
|  | 0.8 nM | 35% |  | |
|  | 1 nM | 27% |  | |
