# Supplementary material for: Quantification of Functionalised Gold Nanoparticle-Targeted Knockdown of Gene Expression in HeLa Cells
Source: PLoS One. 2014 Jun 13;9(6):e99458. doi: 10.1371/journal.pone.0099458 (PMC4057226; doi:10.1371/journal.pone.0099458)
Supplement: Text S1 — Design and optimization of a system for MT gene expression analysis. (DOCX) [file pone.0099458.s009.docx]

**Text S1: Design and optimization of a system for MT gene expression analysis**

To detect and quantify the expression of our target, the human MT-IIa gene (*hMT-IIa*) both at the mRNA level as well as the protein level, we designed and tested hMT-IIa-specific qPCR primers on cDNA samples prepared from untreated cells and those treated with increasing concentrations of inducer CdCl_2_. The *hMT-IIa* gene was expressed at low levels in untreated HeLa cells but upon stimulation with CdCl_2_, the levels of the hMT-IIa mRNA transcript increased significantly (Figure S3). At concentrations above 25 µM CdCl_2_ the levels of *hmTIIa* transcript in the cell decreased, which could be due to toxicity. A concentration of 12.5 µM CdCl_2_ was selected as the working concentration, as it did not result in any changes in the cell morphology, as determined by light microscopy, but provided a reproducibly elevated gene expression level (Figure S4A). The same effect was observed at the protein level (Figure S4B, Table S2A). Knockdown of *hMTIIa* gene expression at these levels would represent a biological effect that could be reliably detected and quantified.
